# Faster Gastrointestinal Transit, Reduced Small Intestinal Smooth Muscle Tone and Dysmotility in the *Nlgn3^R451C^* Mouse Model of Autism

**DOI:** 10.3390/ijms25020832

**Published:** 2024-01-09

**Authors:** Suzanne Hosie, Tanya Abo-Shaban, Kevin Mou, Gayathri K. Balasuriya, Mitra Mohsenipour, Mohammed U. Alamoudi, Rhiannon T. Filippone, Gabrielle T. Belz, Ashley E. Franks, Joel C. Bornstein, Kulmira Nurgali, Elisa L. Hill-Yardin

**Affiliations:** 1 School of Health and Biomedical Sciences, STEM College, RMIT University, Melbourne, VIC 3083, Australiatanya.abo-shaban@rmit.edu.au (T.A.-S.);; 2 Graduate School of Medicine, Kobe University, Kobe 657-8501, Japan; 3 Medical Laboratory Technology Department, Faculty of Applied Medical Sciences, Jazan University, Jazan 45142, Saudi Arabia; 4 Institute for Health and Sport, Victoria University, Melbourne, VIC 3021, Australia; 5 Frazer Institute, The University of Queensland, Brisbane, QLD 4072, Australia; 6 Department of Microbiology, Anatomy, Physiology and Pharmacology, School of Life Sciences, La Trobe University, Melbourne, VIC 3083, Australia; 7 Department of Anatomy and Physiology, University of Melbourne, Melbourne, VIC 3010, Australia; 8 Department of Medicine Western Health, University of Melbourne, Melbourne, VIC 3010, Australia; 9 Regenerative Medicine and Stem Cells Program, Australian Institute for Musculoskeletal Science (AIMSS), Melbourne, VIC 3021, Australia

**Keywords:** autism, gut dysfunction, gut motility, gut transit, mouse model, Neuroligin-3, video-imaging

## Abstract

Individuals with autism often experience gastrointestinal issues but the cause is unknown. Many gene mutations that modify neuronal synapse function are associated with autism and therefore may impact the enteric nervous system that regulates gastrointestinal function. A missense mutation in the *Nlgn3* gene encoding the cell adhesion protein Neuroligin-3 was identified in two brothers with autism who both experienced severe gastrointestinal dysfunction. Mice expressing this mutation (*Nlgn3^R451C^* mice) are a well-studied preclinical model of autism and show autism-relevant characteristics, including impaired social interaction and communication, as well as repetitive behaviour. We previously showed colonic dysmotility in response to GABAergic inhibition and increased myenteric neuronal numbers in the small intestine in *Nlgn3^R451C^* mice bred on a mixed genetic background. Here, we show that gut dysfunction is a persistent phenotype of the *Nlgn3* R451C mutation in mice backcrossed onto a C57BL/6 background. We report that *Nlgn3^R451C^* mice show a 30.9% faster gastrointestinal transit (*p* = 0.0004) in vivo and have 6% longer small intestines (*p* = 0.04) compared to wild-types due to a reduction in smooth muscle tone. In *Nlgn3^R451C^* mice, we observed a decrease in resting jejunal diameter (proximal jejunum: 10.6% decrease, *p* = 0.02; mid: 9.8%, *p* = 0.04; distal: 11.5%, *p* = 0.009) and neurally regulated dysmotility as well as shorter durations of contractile complexes (mid: 25.6% reduction in duration, *p* = 0.009; distal: 30.5%, *p* = 0.004) in the ileum. In *Nlgn3^R451C^* mouse colons, short contractions were inhibited to a greater extent (57.2% by the GABA_A_ antagonist, gabazine, compared to 40.6% in wild-type mice (*p* = 0.007). The inhibition of nitric oxide synthesis decreased the frequency of contractile complexes in the jejunum (WT *p* = 0.0006, *Nlgn3^R451C^ p* = 0.002), but not the ileum, in both wild-type and *Nlgn3^R451C^* mice. These findings demonstrate that changes in enteric nervous system function contribute to gastrointestinal dysmotility in mice expressing the autism-associated R451C missense mutation in the Neuroligin-3 protein.

## 1. Introduction

Autism spectrum disorder (ASD; autism) is a neurodevelopmental disorder diagnosed based on repetitive or restricted activities and deficits in communication and social behaviour. Together with core diagnostic criteria, individuals with autism often experience a range of comorbidities such as increased anxiety [1], irritability [2], and gastrointestinal issues [3,4,5,6,7,8,9,10]. 

More than 1000 gene mutations contribute to ASD [11], and many of them affect neuronal synapse function. Mouse models of autism with gene mutations impacting neuronal synapse function show abnormal brain activity [12,13,14,15,16,17,18,19,20,21,22,23]. Several preclinical models of ASD also show disturbances of enteric neural numbers and caecal size, shifts in the microbiome and gut dysfunction [24,25,26,27,28,29,30,31,32,33]. We propose that mice with mutations impacting the neural circuitry of the brain also show changes in the enteric nervous system within the gastrointestinal tract. However, targeted studies that assess the outputs of neural activity in multiple gut regions are needed to understand the role of these autism-associated mutations in the gut. 

It is well established that the Neuroligin-3 cell adhesion protein is located at neuronal synapses in the brain [34,35,36]. In the enteric nervous system, its mRNA is expressed in both neurons and glia [37,38]. The R451C missense mutation in the Nlgn3 gene encoding was first identified in two brothers diagnosed with ASD [39]. This mutation is well characterized and causes the substitution of an arginine residue to cysteine at position 451 in the Neuroligin-3 protein [40,41]. Mice expressing this mutation show a range of autism-relevant traits, including impaired social communication and repetitive behaviours [14,16,18,42,43]. 

We initially identified a robust aggression phenotype in *Nlgn3^R451C^* mice bred on a mixed genetic background [42]. To verify if this trait was due to the R451C autism-associated mutation as opposed to confounding environmental factors, we backcrossed mice for more than 10 generations onto C57BL/6 mice and assessed them for the persistence of heightened aggression alongside disruptions in the brain’s synaptic activity in the amygdala [14]. We previously reported gastrointestinal dysfunction in both patients and mice (hybrid Sv129/C57BL/6 background) with the R451C missense mutation in the *Nlgn3* gene [24]. Interestingly, we also established that the R451C missense mutation confers a reduction in caecal weight in mice bred on different genetic backgrounds and housed in different environmental conditions [29]. Our recent reports also demonstrate that this mutation causes dysmotility in the caecum [27] and modifies microbial populations in the ileum [44]. These findings suggest that the R451C mutation influences gut function across different environments. However, given that a comprehensive characterization of gut motility in *Nlgn3^R451C^* mice bred on a C57BL/6 genetic background has not yet been conducted, there has been a limited capacity for the comparison of these findings with a substantial proportion of the existing literature describing C57BL/6 intestinal physiology.

Here, we assess the gastrointestinal motility patterns of wild-type (WT) and *Nlgn3^R451C^* mice. We demonstrate changes in intestinal transit in vivo as well as altered gastrointestinal motility patterns in isolated regions of both the small intestine and colon of C57BL/6 *Nlgn3^R451C^* mutant mice. 

## 2. Results

### 2.1. Faster Gastrointestinal Transit in Nlgn3^R451C^ Mice

**Figure 1:**
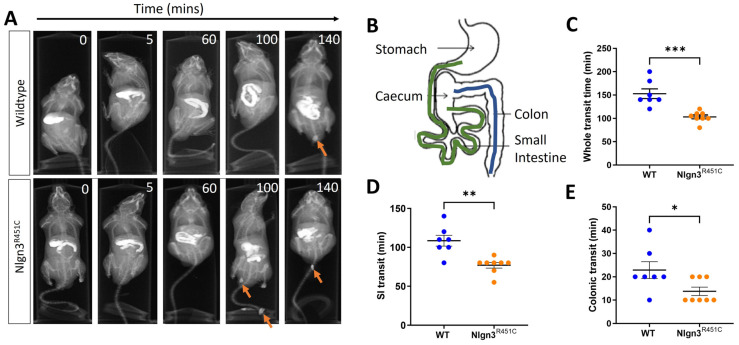
Faster in vivo gastrointestinal transit in *Nlgn3^R451C^* mice. (**A**) Representative X-ray images showing transit of barium sulphate in gastrointestinal tract and faecal pellets (arrows). (**B**) Diagram of mouse gastrointestinal tract indicating regions measured for small intestinal (beginning of duodenum after the pyloric sphincter to the ileum (before ileo-caecal valve)) (green) and colonic (blue) transit. Whole transit time (WT: W = 0.9, *p* = 0.2, *Nlgn3^R451C^*: W = 0.9, *p* = 0.4, Shapiro–Wilk test) (**C**), small intestinal transit time (WT: W = 0.96, *p* = 0.9, *Nlgn3^R451C^*: W = 0.8, *p* = 0.07, Shapiro–Wilk test) (**D**) and colonic transit time (WT: W = 0.9, *p* = 0.2 D’Agostino and Pearson test, *Nlgn3^R451C^*: K2 = 4.9, *p* = 0.08 (Shapiro–Wilk test is inaccurate for several identical data points) (**E**) were compared between WT (*n* = 7) and *Nlgn3^R451C^* (*n* = 8) mice. Data represented as scatterplots of mean ± SEM and individual data points. Student’s *t*-test * *p* < 0.05, ** *p* < 0.01, *** *p* < 0.001. All data passed normality testing.

Gastrointestinal function was assessed in wild-type and *Nlgn3^R451C^* mice using in vivo X-ray imaging. Transit times were compared between *Nlgn3^R451C^* (*n* = 8) and wild-type (WT) mice (*n* = 7) by examining the time taken for BaSO_4_ to travel from the stomach to the anus (whole transit time; WTT), the stomach to the caecum (small intestinal transit time; SITT), and the proximal colon to the anus (colonic transit time; CTT) (Figure 1A,B).

The transit of BaSO_4_ throughout the full length of the gastrointestinal tract was faster in *Nlgn3^R451C^* mice compared to wild-types (WT: 152.9 ± 10.4 min, *Nlgn3^R451C^*: 105.6 ± 4.58 min, *p* = 0.0004; Figure 1C). Regional-specific comparisons indicated that transit times were decreased in both the small and large intestine in *Nlgn3^R451C^* compared to wild-type mice (SITT WT: 108.6 ± 7.05 min, *Nlgn3^R451C^*: 76.88 ± 3.65 min; *p* = 0.0011; Figure 1D) (CTT WT: 22.86 ± 3.60 min, Nlgn3^R451C^: 13.75 ± 1.83 min; *p* = 0.0354, Figure 1E). 

### 2.2. A Reduced Small Intestinal Smooth Muscle Tone in Nlgn3^R451C^ Mice

**Figure 2:**
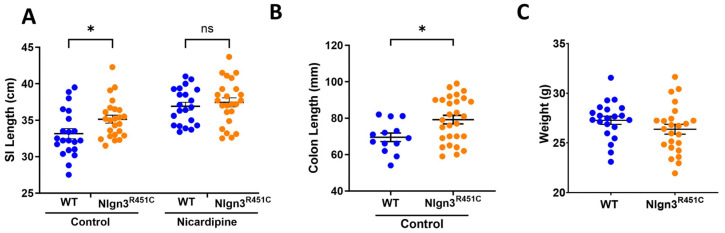
Longer small intestinal and colon length in *Nlgn3^R451C^* mice. (**A**) Wild-type (WT; *n* = 21) and *Nlgn3^R451C^* (*n* = 24) small intestinal (SI) gut length in the active state (control) and after incubation in 100 µM of the muscle relaxant nicardipine (* *p* > 0.05 two-way ANOVA test (variables tested: genotype and nicardipine treatment), Sidak correction). Normality assumptions passed (Shapiro–Wilk normality of residuals test: w = 0.98, *p* = 0.3). (**B**) Increased colon length in *Nlgn3^R451C^* mice (*n* = 28) compared to wild-type mice (*n* = 13) (Student’s *t*-test. * *p* > 0.05). Normality assumptions passed (Shapiro–Wilk normality test WT: w = 0.95, *p* = 0.7; *Nlgn3^R451C^*: w = 0.9, *p* = 0.1). (**C**) No differences were observed between the body weights in a sample of WT (*n* = 21) and *Nlgn3^R451C^* mice (*n* = 24) (Student’s *t*-test. * *p* = 0.17). Normality assumptions passed (Shapiro–Wilk normality test WT: w = 0.97, *p* = 0.6; *Nlgn3^R451C^*: w = 0.98, *p* = 0.9). ns: not statistically significant.

The small intestinal lengths were compared prior to and after incubation in the smooth muscle relaxant and calcium channel blocker, nicardipine, in 21 wild-type and 24 *Nlgn3^R451C^* mice. Initially, compared to wild-types, *Nlgn3^R451C^* mice had longer small intestines (WT: 33.2 ± 0.7 cm; *Nlgn3^R451C^*: 35.2 ± 0.5 cm *p* = 0.044; two-way ANOVA test (factors: genotype and nicardipine treatment), Sidak correction) (Figure 2A). However, following incubation in nicardipine, although the average length of the small intestine increased for both wild-type and *Nlgn3^R451C^* samples, there was no difference in the gut length between genotypes (WT: 36.9 ± 0.5 cm, *Nlgn3^R451C^*: 37.5 ± 0.6 cm; *p* = 0.78; two-way ANOVA test, Sidak correction). These findings suggest that *Nlgn3^R451C^* mice have a reduced small intestinal smooth muscle tone compared to wild-types.

Colon lengths were measured from 21 wild-type and 28 *Nlgn3^R451C^* mice. Similarly to the observations in the small intestine, *Nlgn3^R451C^* mice had longer colons compared to wild-types (WT: 69.5 ± 2.4 mm, *Nlgn3^R451C^*: 79.3 ± 2.3 mm; *p* = 0.014; Student’s *t*-test) (Figure 2B). 

### 2.3. A Reduced Small Intestinal Resting Diameter and Increased Intra-PCC Frequency in Nlgn3^R451C^ Mice

Although it is well established that the R451C mutation in Neuroligin-3 alters neuronal signalling in the brain, little is known about how this mutation impacts the nervous system of the gastrointestinal tract. To examine the impacts on the enteric nervous system (ENS), ENS-regulated intestinal motility patterns were compared in mutant and wild-type mice. Given that different motility patterns, corresponding with known regional-specific physiological roles, occur along the length of the gut, regions of the mouse small intestine (i.e., the ileum and the jejunum) in addition to the colon were assessed to determine region-specific changes and allow for a topographic assessment of the impact of the mutation along the gut.

Historically, much gastrointestinal motility research has focused on the colon, partly due to the difficulty involved in recording the more complex and seemingly irregular motility patterns in the small intestine. The measurement of motility patterns in the small intestine was pioneered in the guinea pig (reviewed in Costa et al., 2021 [45]) using video-imaging techniques [46]. These methods have been translated for utilization in mouse tissue preparations, allowing consistent motility patterns to be recorded and compared in the jejunum of BALB/c and C57BL/6 mice [47]. Alternative methods, such as the utilization of pressure transducers to measure gut contractility, have also been used in the small intestine of mice and have enabled the comparison of motility patterns in the ileum, jejunum and along the entire gastrointestinal tract [48,49,50]. 

In this study, we have built on previous video imaging of the gut motility experiments to record from small intestinal regions of the mouse gastrointestinal tract. Specifically, we identified that increasing luminal pressure above our previously reported colonic recording settings [24,51] enabled consistent recordings of propagating contractile complexes (PCCs) in the mouse jejunum and ileum. In our experimental set up for small intestinal samples, a luminal pressure of 6–6.5 cm H_2_O provided increased consistency in the recorded small intestinal contractile patterns compared to a pressure of approximately 2–3 cm of H_2_O, which is routinely used for colon preparations [51,52,53]. We identified that the level of applied intraluminal pressure is a critical element for the successful recordings of small intestinal motility patterns. This approach, combined with the development of enhanced edge detection software for analysis of the rodent gut contractions in ex vivo preparations [54], enabled a more accurate comparison of motility parameters across experimental groups.

**Figure 3:**
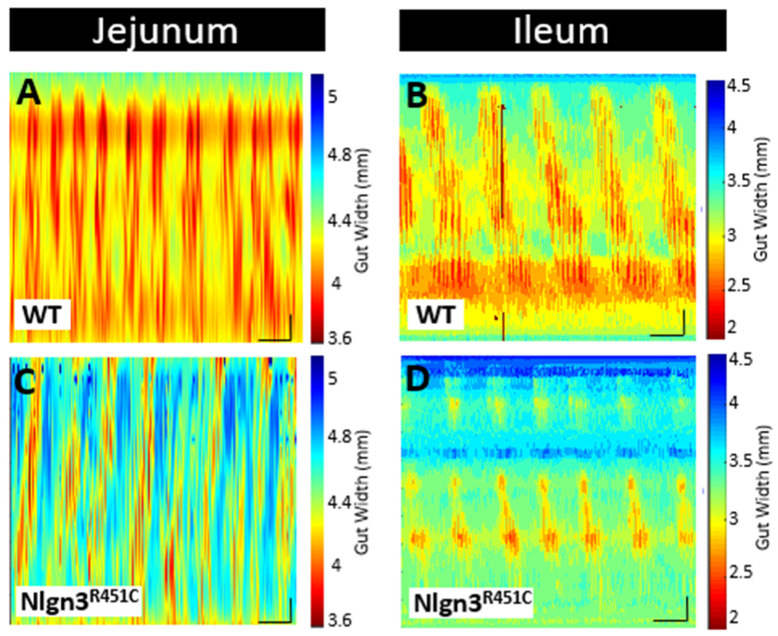
Comparison of small intestinal motility patterns in WT and *Nlgn3^R451C^* mice. Spatiotemporal maps (15 min duration) from (**A**,**C**) jejunum and (**B**,**D**) ileum of WT and *Nlgn3^R451C^* mice. Calibration bar: 10 mm, 200 s.

**Figure 4:**
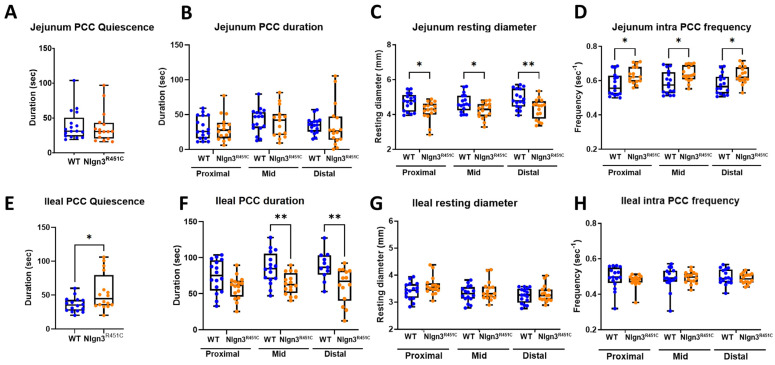
Small intestinal dysmotility in *Nlgn3^R451C^* mice. In the jejunum of *Nlgn3^R451C^* mice, no difference was identified in (**A**) PCC quiescence or (**B**) PCC duration (Shapiro–Wilk normality of residuals assumption passed w = 0.98, *p* = 0.2), however, the (**C**) resting diameter was reduced and the (**D**) intra PCC frequency was faster in all measured regions. In the ileum of the *Nlgn3^R451C^* mice, an increase in (**E**) PCC quiescence and a reduction in (**F**) PCC duration in the mid and distal regions was identified. (**G**) No difference in ileal resting diameter or (**H**) intra PCC frequency was detected between wild-type and *Nlgn3^R451C^* mice. (Student’s *t*-test. * *p* > 0.05, ** *p* < 0.01.)

Here, we used the ex vivo organ bath video-imaging technique [53] to assess motility (i.e., PCCs) in the mouse jejunum and ileum (Figure 3). From the spatiotemporal maps generated, the frequency, quiescence, and velocity of PCCs along the gut segments were compared between wild-type and *Nlgn3^R451C^* mice. These comparisons revealed no differences in PCC frequency or velocity between the wild-type and *Nlgn3^R451C^* jejunum or the ileum. PCC duration was measured at proximal, mid and distal jejunal locations and revealed no differences between genotypes (Figure 4A,B). In the ileum, however, the PCC duration was shorter and a corresponding increase in quiescence was also identified in *Nlgn3 ^R451C^* mice (Figure 4E,F, Table 1). 

To characterise motility patterns in small intestines from wild-type and *Nlgn3^R451C^* mice, we used the enhanced edge detection interface in MATLAB (GutMap, available on request Abo-Shaban et al., 2023 [54]). Specifically, we selected three anatomical locations (proximal, mid and distal) in each small intestinal preparation and measured changes in the gut width over a 15 min recording duration**.** In *Nlgn3^R451C^* mice, the jejunal resting diameter was consistently smaller than in wild-type mice (Table 2, Figure 4C). In contrast, when the jejunal constricted diameter was compared between wild-type and *Nlgn3^R451C^* mice, no difference was observed (Table 2). In the ileum, cross-sectional analysis revealed no genotype difference in either the resting or constricted diameter (Figure 4G, Table 2). 

A power spectrum analysis of the contraction diameter over time enabled the calculation of intra PCC frequency (i.e., the frequency of contractions within a PCC). In the jejunum, intra PCC frequencies measured at the proximal, mid and distal jejunal cross sections were higher in *Nlgn3^R451C^* mice compared to wild-types (Table 2, Figure 4D). We observed similar intra PCC frequencies in the ileum in *Nlgn3^R451C^* and wild-type mice (Table 2, Figure 4H).

Several neurophysiological studies in mice expressing the R451C mutation have focused on GABA neurotransmission, given its major role as an inhibitory neurotransmitter in the brain. A common finding of these studies is that *Nlgn3^R451C^* mice have altered GABAergic neurotransmission in various brain regions [12,18,20]. Therefore, we previously assessed for potential changes in the GABAergic system in the ENS and identified an increased sensitivity of colonic activity to gabazine (a GABA_A_ receptor inhibitor) in *Nlgn3^R451C^* compared to wild-type mice [24]. Within the mouse gastrointestinal tract, the colon expresses significant levels of GABA receptors [55,56]; therefore, this experimental approach seemed appropriate for the colon. In the mouse small intestine, however, the contribution of GABAergic activity to gut function is relatively minor, the expression of GABA receptors is minimal and GABA acts as an excitatory neurotransmitter on enteric neurons [55]. In contrast with the CNS, inhibitory signalling in the intestine occurs at neuromuscular junctions, and is predominantly exerted via nitric oxide in the ENS. In this study, we therefore elected to modulate nitric oxide production and measure small intestinal activity using the video-imaging organ bath assay approach. To inhibit nitric oxide production, we incubated jejunal and ileal segments of the small intestine in the nitric oxide synthase inhibitor, N-nitro-L-arginine (NOLA) and performed video imaging of motility patterns. 

**Figure 5:**
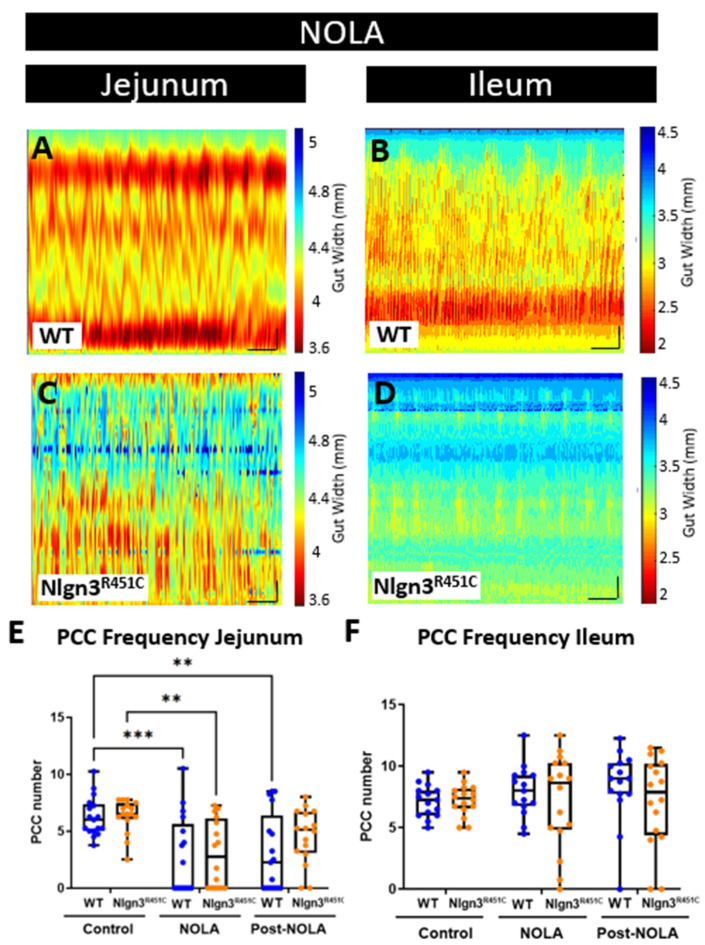
Effect of NOLA on small intestinal motility patterns in wild-type and *Nlgn3^R451C^* mice. Spatiotemporal maps (15 min duration) in WT and *Nlgn3^R451C^* mouse (**A**) jejunum and (**B**) ileum following incubation with NOLA. Average number of PCCs per 15 min before and after incubation with NOLA in (**C**) jejunum and (**D**) ileum in WT and *Nlgn3^R451C^* mice. (**E**) The number of PCCs is reduced following NOLA incubation in the jejunum, However, in the ileum (**F**), PCC numbers persist despite NOLA incubation. Calibration bar: 10 mm, 200 s. Two-way ANOVA test (factors: genotype and NOLA drug), Sidak correction ** *p* < 0.01, *** *p* < 0.001.

In the jejunum, PCC motility patterns were modified in the presence of NOLA (Figure 5A). Specifically, an increase in contractile activity resulted in PCCs being no longer distinguishable in 11 out of 17 of the wild-type and 9 out of 16 of the *Nlgn3^R451C^* gut segments. On average, NOLA reduced the number of defined PCCs per 15 min in the jejunum in both WT and *Nlgn3^R451C^* (WT (control): 6.3 ± 0.4, WT (NOLA): 2.7 ± 0.8; *p* = 0.0006; *Nlgn3^R451C^* (control): 6.3 ± 0.4, *Nlgn3^R451C^* (NOLA): 3.0 ± 0.7 PCCs; mean ± SEM, *p* = 0.002 (two-way ANOVA (factors: genotype and NOLA effect), Sidak correction); Figure 5A,C,E). In contrast, NOLA had no significant effect on ileal PCC frequency per 15 min (WT (control): 7.2 ± 0.3, WT (NOLA): 7.5 ± 0.7; *p* = 0.94, *Nlgn3^R451C^* (control): 7.2 ± 0.3, *Nlgn3^R451C^* (NOLA): 7.2 ± 1 PCCs; mean ± SEM, *p* > 0.99). In addition, NOLA did not alter ileal PCC velocity (WT (control): 0.4 ± 0.1, *Nlgn3^R451C^*: (control): 0.4 ± 0.06; *p* = 0.9, WT (NOLA): −0.1 ± 0.05, *Nlgn3^R451C^* (NOLA): −0.13 ± 0.1 mm/s mean ± SEM, *p* = 0.9) or duration. There were no changes in PCC duration in the proximal ileum under baseline conditions (WT (control): 74.1 ± 5.7, *Nlgn3^R451C^* (control): 58.5 ± 4 s *p* = 0.09, mean ± SEM). 

However, upon NOLA application in the same group of mice, in *Nlgn3^R451C^* mice, the PCC clusters were distinguishable and retained the form seen in baseline heatmaps, whereas WT PCCs were disrupted. During NOLA application, in *Nlgn3^R451C^* mice, the PCC duration in the proximal ileum increased (WT (NOLA): 3.4 ± 0.1, *Nlgn3^R451C^* (NOLA): 24.8 ± 5 s *p* < 0.0001, mean ± SEM). In contrast, in the mid and distal regions of the ileum, the PCC duration decreased (mid ileal region: WT (control): 85.2 ± 6, *Nlgn3^R451C^* (control): 63.4 ± 4 s *p* = 0.009; distal ileum region WT (control): 88.3 ± 6, *Nlgn3^R451C^* (control): 61.4 ± 6.5 s *p* = 0.004, mean ± SEM). However, following the super-fusion of NOLA, the PCC duration increased in the mid region only (mid ileum region: WT (NOLA): 3.2 ± 0.09, *Nlgn3^R451C^* (NOLA): 28.0 ± 4 s *p* < 0.0001). There was no genotype difference in the PCC duration in the distal ileum region during NOLA application (WT (NOLA): 3.5 ± 3, *Nlgn3^R451C^* (NOLA): 10.8 ± 6 s *p* = 0.3, mean ± SEM) (Figure 5B,D,F).

### 2.4. The Increased Short Colonic Contractions in Nlgn3^R451C^ Mice

Colonic motility was compared using the ex vivo organ bath video-imaging technique in 17 wild-type and 14 *Nlgn3^R451C^* mice. Previous findings examining colonic motility in *Nlgn3^R451C^* mice bred on a mixed-background strain showed a decrease in the frequency of colonic migrating motor complexes (CMMCs) in the presence of the GABA_A_ receptor antagonists, gabazine and bicuculline [24]. Our previous study [24] showed that the decrease in CMMC frequency was more pronounced in response to gabazine. Therefore, to tease out potential differences in motility patterns in WT and *Nlgn3^R451C^* C57BL/6 colons, we similarly applied gabazine to the organ bath. For these experiments examining colonic motility, gabazine was utilised as opposed to NOLA to determine if findings in the brain were replicated to some extent in the ENS. In addition, as outlined previously, the mouse colon is a more suitable region to test GABA_A_ receptor responses compared to the small intestine due to the greater expression of GABA receptors in the mouse colon compared to the small intestine [57]. 

**Figure 6:**
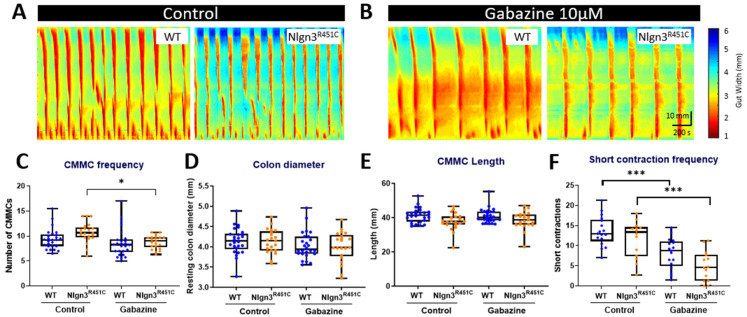
Gabazine reduces the proportion of short colonic contractions in *Nlgn3^R451C^* mice. Representative spatiotemporal maps of WT and *Nlgn3^R451C^* colonic activity in (**A**) control conditions and (**B**) after administration of 10 µM gabazine to the organ bath. (**C**) Effect of gabazine on CMMC frequency in WT and *Nlgn3^R451C^* mice. No changes were observed for (**D**) resting colon diameter or (**E**) CMMC length. (**F**) The short contraction frequency was reduced in both WT and *Nlgn3^R451C^* colons; WT *n* = 18; *Nlgn3^R451C^ n* = 14. Calibration bar: 10 mm, 200 s. One-way ANOVA Holm–Sidak multiple comparisons test. * *p* < 0.05, *** *p* < 0.001.

The properties of CMMCs in WT and *Nlgn3^R451C^* were compared in control conditions and in the presence of the GABA_A_ receptor antagonist, gabazine (Figure 6A,B). Although the mean CMMC frequency per 15 min was higher in *Nlgn3^R451C^* under control conditions, this did not reach statistical significance (WT control: 9.4 ± 0.4, *Nlgn3^R451C^* control: 10.7 ± 0.4, mean ± SEM, *p* = 0.07). Gabazine reduced CMMC frequency in both WT and *Nlgn3^R451C^* colons; however, this was statistically significant in *Nlgn3^R451C^*, but not WT colons (WT control: 9.4 ± 0.4, gabazine: 8.6 ± 0.5, mean ± SEM, *p* = 0.51; *Nlgn3^R451C^* control: 10.7 ± 0.4, gabazine: 8.8 ± 0.3, mean ± SEM, *p* = 0.03; Figure 6C). There were no differences in resting colon diameter (Figure 6D) or CMMC length (Figure 6E) in control conditions or in the presence of gabazine. 

We also measured short anal contractions which are defined as contractions that extend less than 50% of the full length of the colon segment and appear in the distal region of the colon. Under control conditions, WT and *Nlgn3^R451C^* colons displayed similar short anal contraction frequencies (control WT: 13.6 ± 0.9, *Nlgn3^R451C^*: 11.7 ± 1.2, mean ± SEM, *p ≥* 0.99). In the presence of gabazine, the short contraction frequency was reduced in both WT and *Nlgn3^R451C^* colons. Although *Nlgn3^R451C^* mice had fewer short anal contractions compared to WT, this was not statistically different (gabazine WT: 8.1 ± 0.9, gabazine *Nlgn3^R451C^*: 5.0 ± 1.0, mean ± SEM, *p* = 0.64; Figure 6F). When analysing the number of short contractions as a percentage of total colonic contractions, *Nlgn3^R451C^* mice had a lower percentage of short anal contractions compared to WT (gabazine WT: 48.2% ± 3.9, gabazine *Nlgn3^R451C^*: 32.1% ± 5.2, mean ± SEM, *p* = 0.016). 

## 3. Discussion

Here, we confirm that C57BL/6 mice with the R451C mutation share a similar phenotype consisting of faster gastrointestinal transit and increased sensitivity to the GABA_A_ receptor antagonist, gabazine, as previously reported in *Nlgn3^R451C^* mixed-background mice. In this study, we additionally examined whole gut transit, smooth muscle tone and intestinal motility patterns in the ileum, jejunum and colon of wild-type and *Nlgn3^R451C^* mice. 

We observed faster full-length gastrointestinal transit in *Nlgn3^R451C^* mice. Transit times were also reduced in the small intestine and colon, suggesting that the impact of the R451C mutation on gastrointestinal transit is not restricted to a specific gut region. Notably, children with ASD are seven times more likely to have diarrhoea than neurotypical children [58]; therefore a reduction in transit time in this mouse model may provide a clinically relevant tool for future mechanistic analyses. Our previous findings in *Nlgn3^R451C^* mice examined animals bred on a mixed genetic background and showed faster transit in the small intestine alongside elevated numbers of nNOS neurons in the jejunum [24]. Interestingly, these findings are in contrast with another report in mice with a faster gastrointestinal transit at day 3 post-treatment with an anti-cancer agent, 5-fluorouracil (5-FU), associated with a reduction in myenteric nNOS neurons but slower gastrointestinal transit at day 14 post-5-FU treatment [59]. A slower gastrointestinal transit time associated with an increased proportion of myenteric nNOS neurons was observed following 14 days of treatment with another chemotherapeutic drug, oxaliplatin [60]. The differential effects on gastrointestinal transit and enteric nNOS neurons might be due to the differences in the mechanisms of action of various compounds, with 5-FU causing intestinal inflammation and oxaliplatin causing direct toxicity. Moreover, morphological and functional changes in other types of enteric neurons could affect gastrointestinal transit, as was observed in irinotecan-treated mice, which had increased intestinal transit and acute secretory diarrhoea at day 3 post-treatment associated with an increased number of cholinergic neurons and fibres [61]. Chronic intestinal inflammation causes an indiscriminate loss of enteric neurons and an increased colonic transit time [62,63,64]. Alterations of intestinal transit might result from the modulation of both extrinsic (sympathetic and parasympathetic) and intrinsic innervation of the gastrointestinal tract, changes in intestinal smooth muscle tone, morphological and functional changes in interstitial cells of Cajal and neuromuscular transmission [65,66]. Previous examples of inflammatory dysfunction that may impact gut motility in preclinical models of ASD include our previous report in which we identified changes in Iba-1 macrophage density and morphology in gut-associated lymphoid tissue in the caecum of *Nlgn3^R451C^* mice under control conditions [29]. Further exploration is needed to determine the mechanism underlying the increased transit in *Nlgn3^R451C^* mice but given that individuals with ASD often experience alternating constipation and diarrhea, the presence of this clinically relevant phenotype provides a strong rationale for the use of this model to investigate the causes of gastrointestinal symptoms experienced in ASD. 

Surprisingly, we found that *Nlgn3^R451C^* mice have longer small intestines and colons but that this was abolished in small intestinal preparations following incubation in the muscle relaxant, nicardipine. The identification of longer colons in *Nlgn3^R451C^* mice bred on a mixed genetic background under control conditions suggests that muscle tone is similarly reduced across different gut regions and genetic backgrounds. We speculate that *Nlgn3^R451C^* mice have a decreased small intestinal longitudinal and circular smooth muscle tone that may contribute to the increased colon length we observed in the mutants. Although the biological mechanism underlying this increased gut length is unknown, the R451C mutation might potentiate the inhibitory role of NO to reduce smooth muscle activity under baseline conditions. It is well established that Neuroligin-3 interacts with the key enzyme for NO production (neuronal nitric oxide synthase; NOS) [67,68,69]. Furthermore, we previously showed an increase in the number of nNOS neurons in the jejunal myenteric plexus of *Nlgn3^R451C^* mice [26] which could boost the production of neuronal NO in these mice. To determine if this is the case, further analyses of wild-type and *Nlgn3^R451C^* gastrointestinal tissue at the gene and protein expression level are required. 

*Nlgn3^R451C^* mice have altered small intestinal motility as assessed via ex vivo organ bath assays. We observed that the diameter of the *Nlgn3^R451C^* jejunum was more constricted under resting conditions. Given that we observed an increase in small intestinal length in these mice, this elongation (presumably involving longitudinal smooth muscle relaxation) may occur simultaneously with an elevated contraction of the circular smooth muscle [70]. *Nlgn3^R451C^* mice also showed an increase in intra PCC frequency in the jejunum. This high frequency component of the spatiotemporal map has previously been identified to oscillate at a similar frequency to slow waves in the jejunum [47,50]. Slow waves are a rhythmical depolarisation of the smooth muscle and are driven by the pacemaker activity of the interstitial cells of Cajal (ICC) [71,72]. The pacemaker ICC are associated with the myenteric plexus and are electrically coupled to each other and to the smooth muscle [73]. In the small intestine, the amplitude of slow waves is not sufficient to trigger smooth muscle action potentials and muscle contraction, therefore an additional neurogenic component is required for typical motility patterning. In *Nlgn3^R451C^* mice, the observed increase in intra PCC frequency may be a result of altered synaptic activity in the enteric neuronal pathway that influences slow wave generation, perhaps via the intramuscular population of ICC, which are innervated. 

In the *Nlgn3^R451C^* mouse ileum, the motility phenotype differed from the jejunum. We found that the quiescent period between ileal PCCs was increased, along with corresponding shorter PCC durations in the proximal, mid and distal regions of the ileum. Potentially, the reduction in the overall PCC/churning time may cause the general flow of gut content to be less inhibited and, therefore, have a reduced/faster transit time. In turn, less PCC/churning time could also subtly influence the rate of the absorption of nutrients and water from the gastrointestinal content in these mice. This aligns with our data showing faster intestinal transit using X-ray imaging. How the R451C mutation influences the duration of PCCs is unclear, although this could involve an imbalance of neurogenic excitation and inhibition in this region of the gastrointestinal tract. 

In this study, we compared regional gastrointestinal motility patterns in the small intestine using an enhanced software interface in MATLAB, including a novel edge detection algorithm for the video imaging of gut contractile activity in fresh tissue preparations [54]. Although the development of the software interface and optimization of experimental conditions (including the level of applied pressure to the lumen of the gastrointestinal tissue sample) improved our ability to record and analyse motility patterns in the mouse jejunum and ileum, the interpretation of these complex small intestinal motility patterns continues to be developed. Further investigations are needed to understand how in vivo gastrointestinal transit data correlates with in vitro motility patterns. For example, some of our ex vivo findings (i.e., an increase in intra PCC frequency in the jejunum and a higher speed of PCCs in the ileum) appear to correlate with the faster transit seen in vivo in *Nlgn3^R451C^* mice via X-ray imaging, but potential correlations across the preparations for other parameters, such as the observed increase in baseline jejunal constriction in *Nlgn3^R451C^* mice, are less straightforward in terms of our understanding of how they contribute to physiological profiles in intact animals. 

We found reduced numbers of short contractions occurring in the distal colon of *Nlgn3^R451C^* mice in response to the GABA_A_ receptor antagonist, gabazine, suggesting an increased sensitivity or potentially fewer functional GABA_A_ receptors in the *Nlgn3^R451C^* mouse colon. Currently, the precise function of distal short contractions in the mouse colon is unknown. Future studies may assist in gaining further insights into their physiological role/s; however, until then they provide a useful comparison tool for colonic motility patterning across mouse models of a range of clinically relevant disorders [74]. The results of the current study support our previous findings in the *Nlgn3^R451C^* mouse model bred on a mixed-background strain [24] and provide further evidence for GABA_A_-mediated colonic dysmotility in mice expressing the R451C mutation in Neuroligin-3. Overall, these findings suggest that GABA_A_ receptors may be involved in mediating the initiation of colonic short contractions.

In all, these findings suggest that the autism-associated R451C missense mutation in the *Nlgn3* gene impacts the structure and function of the mouse gastrointestinal tract. The presence of the Neuroligin-3 cell adhesion protein in the brain is well established [12,16,20,23,35,38], and *Nlgn3* is also expressed in the mouse gastrointestinal tract [24,44,52]. In fact, we have recently detected *Nlgn3* mRNA in most enteric neuronal and glial populations in the mouse ileum [37]. It is possible that the regional differences in motility we observed are caused by different expression levels of *Nlgn3* in the jejunum, ileum and colon; however, this remains to be determined.

## 4. Materials and Methods

### 4.1. Mice

*Nlgn3^R451C^* mixed background (Sv129/ImJ/C57BL/6) mice were initially obtained from The Jackson Laboratory (Bar Harbour, ME, USA), and subsequently backcrossed with C57BL/6 mice for more than 10 generations and maintained on the C57BL/6 genetic background. 

Mice used for X-ray imaging experiments were 14 weeks old and imported from the Florey Institute (Parkville, VIC, Australia) to the RMIT Animal Facility (RAF; Bundoora, VIC, Australia). Mice used for measurements of gut length or intestinal motility experiments were aged between 8–14 weeks and euthanized by cervical dislocation. All experimental procedures undertaken on the mice were approved by the RMIT University Animal Ethics Committee (AEC 1727). 

### 4.2. Whole Body X-ray Imaging 

The X-ray imaging technique was undertaken as detailed by McQuade et al., 2016 [59]. In brief, prior to X-ray imaging, mice underwent an acclimatization period of 1 week where they were conditioned to oral gavage by having a plastic gavage tip (Instech Laboratories Inc., Plymouth Meeting, PA, USA; 20 ga × 30 mm) inserted at least three times for each animal, with at least 24 h between each conditioning session. Mice were also conditioned for restraint by placing a transparent plastic restraint tube (Terumo Corporation, Tokyo, Japan; 50 mL) into the mouse cages at least 24 h prior to the X-ray experiment.

For the X-ray procedure, 0.2 mL of barium sulphate (BaSO_4_; X-OPAQUE-HD, 2.5 g/mL) was administered to mice via oral gavage. Mice were then immobilized in the prone position inside a restraint tube and were X-ray imaged using Fujifilm cassettes (24 × 30 cm) and a HiRay Plus Porta610HF X-ray apparatus (JOC Corp, Kanagawa, Japan; 50 kV, 0.3 milliampere-second, 60 ms exposure time). X-rays were captured immediately after administration of BaSO_4_, every 5 min for the first hour, every 10 min for the second hour, then every 20 min afterwards, until the first faecal pellet containing BaSO_4_ was expelled. Animals were free to move between X-rays and were closely monitored during and after all procedures. Images were developed via a Fujifilm FCR Capsula XLII and analysed using eFilm 4.0.2 software. 

The rate of gastrointestinal transit was measured by inferring the time (in minutes) it took for BaSO4 to reach different regions of the gut. The transit times of interest were from the stomach to the caecum (small intestinal time; SITT), proximal colon to the anus (colonic transit time; CTT), and the stomach to the anus (whole transit time; WTT). Only mice that produced faecal pellets containing BaSO_4_ as visualised in the X-ray images were included in the study (out of 16 mice, 1 *Nlgn3^R451C^* mouse was excluded for not producing pellets in the time frame).

### 4.3. Gut Length Measurement

The entire gastrointestinal tract was carefully separated from the mesentery and removed from culled adult male WT and *Nlgn3^R451C^* mice. Using dissecting scissors, the small intestine from the pylorus to the ileocaecal junction was detached from the stomach and caecum, laid onto a flat surface and measured using a tape measure. A digital image of the tissue was then acquired. To assess the maximal length of the tissue, a proportion of the small intestinal gut preparations were then placed in a glass beaker containing the L-type calcium channel blocker, nicardipine (Sigma-Aldrich, Castle Hill, NSW, Australia) (3 mM) for 20 min. The small intestinal preparations were then removed from the nicardipine solution, re-measured and re-imaged for comparison.

Colons (proximal colon to rectum) from WT and *Nlgn3^R451C^* mice were dissected, and the surrounding mesentery was removed. Colon preparations were attached to an organ bath using a small insect pin (Australia Entomology supplies, AUS). Tissue preparations were positioned adjacent to a printed ruler located in the base of the organ bath and a digital image was acquired. Colon lengths were measured using ImageJ Fiji software v2.9.0 and were calibrated according to the ruler measurements. 

### 4.4. Video Imaging Intestinal Motility 

Adult male WT and Nlgn3R451C mice (aged 8–14 weeks) were culled by cervical dislocation and the gastrointestinal tract, from the stomach to the rectum, was removed. Jejunal segments measuring approximately 6 cm in length were taken from a position located 5 cm from the stomach. Ileal tissue measuring approximately 5 cm in length was removed from the location of the ileo-caecal junction. 

All intestinal motility experiments were carried out using our previously described video-imaging technique approach for recording colonic motility in mice [53]. As previously reported [53], we applied intraluminal pressure to gastrointestinal tissue preparations in the organ bath as measured via the height of the fluid meniscus on the organ bath apparatus. Meniscus height was determined by subtracting the height of the organ bath from the fluid meniscus height of the front pressure outlet tube and the fluid meniscus height of the back pressure outlet tube. 

For the duration of the video-imaging experiment, tissue was continuously super-fused with 1x Krebs solution (composition, mM: 118 NaCl, 4.76 KCl, 0.99 NaH_2_PO_4_.2H_2_O, 1.20 MgSO_4_.7H_2_O, 2.5 CaCl_2_.2H_2_O, 11.1 D-glucose, 25 NaHCO_3_). Krebs solution was bubbled with carbogen (95% O_2_ and 5% CO_2_) (BOC Limited, North Ryde, NSW, Australia), and the temperature of the organ bath was maintained between 33 and 35 °C.

Experimental protocols consisted of an initial equilibration period (2 × 15 min) followed by control recordings (4 × 15 min), during which time the tissue preparations were incubated in Krebs physiological solution. Subsequently, either 100 µM N-nitro-L-arginine (NOLA) (Sigma-Aldrich, Castle Hill, NSW, Australia) or 10 µM gabazine (Sigma-Aldrich, Castle Hill, NSW, Australia) (dissolved in 1× Krebs solution) was infused into the tissue bath and recordings were made for 1 h (4 × 15 min). Finally, a washout period was conducted and recordings were captured (4 × 15 min) during which the super-fusion was replaced with 1x Krebs solution. Reagent details for organ bath perfusion: NaCl (Chem-Supply, SA046-5KG, Gillman SA, Australia); KCl (Ajax Finechem, AJA383-500G, Wollongong, NSW, Australia); NaH_2_PO_4_.2H_2_O (Chem-Supply, SA328-500G); MgSO_4_.H_2_O (Chem-Supply, MA048-500G); CaCl_2_.2H_2_O (Ajax Finechem, 127-500G); NaHCO_3_ (Chem-Supply, SA001-5KG); D-Glucose anhydrous (Chem-Supply, GA018-500G). 

Gut contractile activity was recorded at a frame rate of 30 Hz using a Logitech camera (Quickcam Pro 9000)mounted above the organ bath. Spatiotemporal heatmaps in the colon and small intestine, respectively, were generated using in-house software (Scribble v2.10 [53]) or the GutMap MATLAB software interface [54]. A subset of these datasets were analyzed using both Scribble v2.10 and GutMap and yielded identical results. The software performed edge detection, converting an image of the gut tissue into a silhouette from which the number of vertical pixels for each horizontal pixel was counted. A spatiotemporal heatmap was generated by plotting the diameter of the small intestine segment as a function of space and time.

### 4.5. The Measurement of PCC Parameters: Frequency, Duration, Length and Velocity

**Figure 7:**
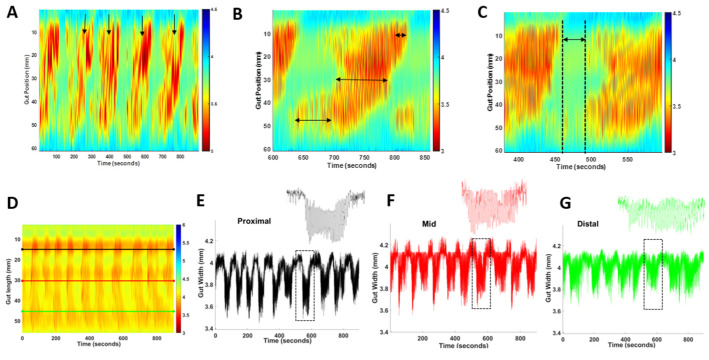
PCC analysis method. (**A**) Frequency was calculated by counting each PCC (arrows) that occurred during each 15 min recording. PCC duration was measured at the proximal, mid and distal location shown by arrows on map (**B**). (**C**) Quiescence was measured as the time interval between PCCs (as indicated by the arrow on the map). (**D**) Representative spatiotemporal map from a wild-type mouse indicating location of proximal (black), mid (red) and distal (green) gut width measurements. (**E**–**G**) Gut width measurements against time for the anatomical locations indicated in A; inserts represent enlarged single PCCs (dashed boxes).

Small intestinal heatmaps were analysed in MATLAB R2021b using in-house software GutMap. The frequency of propagating contractile clusters (PCCs) was determined by manually counting contractile clusters that propagated in a consistent direction for more than 50% of the gut length segment (Figure 7A). For each PCC, the duration was determined by measuring the time at the start and end of the PCC and subtracting the difference. To ascertain the maximum length of the PCC, measurements were taken from the most proximal and distal regions of the PCC and the difference was calculated (Figure 7B). The PCC velocity was calculated using GutMap software. To measure the rest period between the PCCs (i.e., quiescence), the time duration was measured between the end of the PCC contraction and the start of the next PCC contraction (Figure 7C). Gut width was plotted against time for each of the selected regions and the average resting diameter and constricted diameter were compared for WT and *Nlgn3^R451C^* mice (Figure 7D–G). 

### 4.6. Regional PCC Parameters

Within a single PCC, the duration of individual contractions varied along the length of the tissue; therefore, a single PCC was divided into three types of contractions for analysis (i.e., proximal short, full-length and distal short contractions). Proximal short contractions were defined as short contractions which contracted between the proximal and middle region of the small intestinal preparation. Full-length contractions were defined as contractions which propagated the full length of the gut tissue and, lastly, distal short contractions were defined as contractions occurring between the mid region and distal end of the small intestinal preparation. Contraction velocity for each sample was measured using the in-house-created GutMap software v.1.0 as an average value calculated from the velocity of 6 randomly selected contractions. 

### 4.7. Maximum and Minimum Gut Diameter and Slow Wave Frequency

To measure the maximum and minimum diameter of the gut tissue, measurements of gut width were plotted from the spatiotemporal map against time at selected proximal, middle and distal locations (Figure 3A). The same gut measurements were analysed using the Fast Fourier Transform algorithm resulting in a histogram whereby the peak described the most common frequency between contractions. The frequency of the peaks per intra PCC cluster was measured to provide the slow wave frequency (intra PCC frequency) for each of the selected gut regions. The average intra PCC frequency was calculated across tissue replicates. 

### 4.8. Statistical Analyses

Transit times from X-ray imaging experiments were compared using a Student’s unpaired *t*-test (Mean ± SEM). Small intestinal length measurements were compared using a two-way ANOVA test with Sidak correction (Mean ± SEM), given that there were four groups including WT and *Nlgn3^R451C^* samples treated with Nicardipine. Colon lengths and mouse body weights were compared using a Student’s unpaired *t*-test (Mean ± SEM). PCC frequency, length, velocity, duration, resting diameter and inter PCC frequency were compared using a two-way ANOVA test with Sidak correction. The intra PCC cluster frequency was compared using a Mann–Whitney U test with Holm–Sidak correction. Two-way ANOVA tests were used for datasets comparing genotype and drug effects (i.e., nicardipine and NOLA). CMMC frequency, colon diameter and colon length, as well as short anal contractions were compared using a one-way ANOVA Holm–Sidak multiple comparisons test. All datasets were screened for normality (Shapiro–Wilk test, except for colonic transit data which was tested using the D’Agostino and Pearson test, given that several of these values were identical and Shapiro–Wilk test is inaccurate for multiple identical data points) and analysed accordingly (either ANOVA or *t*-tests for normal distributions and Mann–Whitney U test for datasets that did not satisfy normality testing) using Prism v9.5.1 software. 

## 5. Conclusions

Our findings indicate that mice expressing an autism-associated mutation in the gene encoding the Neuroligin-3 protein display gut dysfunction. We showed that gastrointestinal transit along the full length of the gut was faster in *Nlgn3^R451C^* mice compared to wild-types. Additionally, when the transit times were assessed for multiple gastrointestinal segments in these mice, we consistently found that the transit in each gastrointestinal region was faster in mutant mice. *Nlgn3^R451C^* mice have longer small intestines and colons compared to wild-type mice which could indicate an increased relaxation of smooth muscle, potentially resulting from enteric neural dysfunction. In *Nlgn3^R451C^* mice, the jejunal resting diameter was more constricted than in wild-types. *Nlgn3^R451C^* mice had altered intestinal motility with faster intra PCC frequency in the proximal, mid and distal sections of the jejunum when compared to wild-type mice. In the ileum, PCC durations were shorter and there was a longer quiescence period between PCCs. Additionally, colons from *Nlgn3^R451C^* mice showed an increased sensitivity to the GABA_A_ receptor antagonist gabazine. 

Characterising the expression profile of *Nlgn3* within the enteric nervous system, including within the specific functional subtypes of enteric neurons is crucial to understanding the impact of the R451C mutation on gut function and is an ongoing area of investigation. These findings have potential implications for enhancing our understanding of the underlying causes of GI disorders associated with autism and for identifying therapeutic targets.

## Figures and Tables

**Table 1:** Small intestinal motility parameters from the jejunum and ileum in wild-type and *Nlgn3^R451C^* mice.

Small Intestinal Motility Parameter	Jejunum			Ileum		
	WT n = 17	*Nlgn3^R451C^* n = 16	Normality Passed?	WT n = 16	*Nlgn3^R451C^* n = 16	Normality Passed?
**Full length PCC Frequency** **(No. of PCCs/15 min)**	6.3 ± 0.4	6.3 ± 0.4	Yes. WT: w = 0.9, *p* = 0.3. *Nlgn3^R451C^*: w = 0.9, *p* = 0.051	7.2 ± 0.3	7.2 ± 0.3	Yes. WT: w = 0.99, *p* > 0.99. *Nlgn3^R451C^*: w = 0.97, *p* = 0.8
**Full length PCC Quiescence (s)**	39.4 ± 5.3	37.6 ± 5.8	Yes. WT: w = 0.9, *p* = 0.2. *Nlgn3^R451C^*: w = 0.9, *p* = 0.3	35.8 ± 2.7	**54.3** ± **6.8** *	Yes. WT: w = 0.96, *p* = 0.6. *Nlgn3^R451C^*: w = 0.9, *p* = 0.06
**Full length PCC Velocity (mm/s)**	0.5 ± 0.02	0.6 ± 0.06	Yes. WT: w = 0.9, *p* = 0.3. *Nlgn3^R451C^*: w = 0.9, *p* = 0.4	−0.3 ± 0.1	−0.4 ± 0.2	Yes. WT: w = 0.9, *p* = 0.4. *Nlgn3^R451C^*: w = 0.98, *p* = 0.9
**Proximal region PCC duration (s)**	30.9 ± 4	30.8 ± 4.5	Yes. WT: w = 0.9, *p* = 0.1. *Nlgn3^R451C^*: w = 0.96, *p* = 0.7	74.1 ± 5.7	58.5 ± 4	Yes. WT: w = 0.9, *p* = 0.2. *Nlgn3^R451C^*: w = 0.96, *p* = 0.7
**Mid region PCC duration (s)**	41.6 ± 4.3	39.4 ± 5.6	Yes. WT: w = 0.9, *p* = 0.1. *Nlgn3^R451C^*: w = 0.9, *p* = 0.4	85.2 ± 5.6	**63.4** ± **3.7** ^†^	Yes. WT: w = 0.98, *p* = 0.98. *Nlgn3^R451C^*: w = 0.96, *p* = 0.7
**Distal region PCC duration (s)**	34.5 ± 2.8	34.4 ± 7.8	Yes. WT: w = 0.97, *p* = 0.7. *Nlgn3^R451C^*: w = 0.9, *p* = 0.4	88.3 ± 6.1	**61.4** ± **6.5** ^†^	Yes. WT: w = 0.99, *p* = 0.99. *Nlgn3^R451C^*: w = 0.9, *p* = 0.2

Mean ± SEM, genotype comparisons; * *p* < 0.05 ^†^ *p* < 0.01. Shapiro–Wilk normality test performed.

**Table 2:** Regional small intestinal motility parameters from jejunum and ileum in wild-type and *Nlgn3 ^R451C^* mice.

Small Intestinal Motility Parameters		Jejunum			Ileum		
		WT *n* = 17	*Nlgn3^R451C^* *n* = 16	Normality Passed?	WT *n* = 16	*Nlgn3^R451C^* *n* = 16	Normality Passed?
**Resting diameter (mm)**	Prox	4.7 ± 0.1	**4.2** ± **0.1** *	Yes. WT: w = 0.9, *p* = 0.3. *Nlgn3^R451C^*: w = 0.9, *p* = 0.1	3.4 ± 0.1	3.6 ± 0.1	Yes. WT: w = 0.98, *p* = 0.96. *Nlgn3^R451C^*: w = 0.9, *p* = 0.07
	Mid	4.7 ± 0.1	**4.2** ± **0.1** *	Yes. WT: w = 0.9, *p* = 0.3. *Nlgn3^R451C^*: w = 0.96, *p* = 0.6	3.3 ± 0.1	3.4 ± 0.1	Yes. WT: w = 0.98, *p* = 0.9. *Nlgn3^R451C^*: w = 0.9, *p* = 0.07
	Distal	4.9 ± 0.1	**4.3** ± **0.1** ^†^	Yes. WT: w = 0.9, *p* = 0.4. *Nlgn3^R451C^*: w = 0.95, *p* = 0.5	3.2 ± 0.1	3.3 ± 0.1	Yes. WT: w = 0.9, *p* = 0.2. *Nlgn3^R451C^*: w = 0.9, *p* = 0.1
**Constricted diameter (mm)**	Prox	3.9 ± 0.1	3.5 ± 0.1	Yes. WT: w = 0.9, *p* = 0.3. *Nlgn3^R451C^*: w = 0.9, *p* = 0.1	2.7 ± 0.1	2.9 ± 0.1	Yes. WT: w = 0.95, *p* = 0.6. *Nlgn3^R451C^*: w = 0.9, *p* = 0.06
	Mid	3.7 ± 0.1	3.5 ± 0.1	Yes. WT: w = 0.9, *p* = 0.3. *Nlgn3^R451C^*: w = 0.95, *p* = 0.5	2.5 ± 0.1	2.7 ± 0.1	Yes. WT: w = 0.98, *p* = 0.9. *Nlgn3^R451C^*: w = 0.9, *p* = 0.2
	Distal	4.0 ± 0.1	**3.6** ± **0.1** *	Yes. WT: w = 0.98, *p* = 0.95. *Nlgn3^R451C^*: w = 0.9, *p* = 0.4	2.5 ± 0.1	2.7 ± 0.1	Yes. WT: w = 0.9, *p* = 0.3. *Nlgn3^R451C^*: w = 0.9, *p* = 0.1
**Intra PCC frequency (sec^−1^)**	Prox	CI: 0.5–0.7	**CI: 0.6**–**0.7** *	No. w = 0.95, *p* = 0.0002	CI: 0.3–0.6	CI: 0.4–0.5	No. w = 0.91, *p* < 0.0001
	Mid	CI:0.5–0.7	**CI: 0.6**–**0.7** *		CI: 0.3–0.6	CI: 0.4–0.6	
	Distal	CI: 0.5–0.7	**CI: 0.5**–**0.6** *		CI: 0.4–0.6	CI: 0.4–0.5	

Mean ± SEM, genotype comparisons; confidence intervals (CI) * *p* < 0.05 ^†^ *p* < 0.01. Shapiro–Wilk normality test performed.

## Data Availability

Data and the MATLAB analysis software interface are available on request.
